# Barriers to the successful practice of chronic kidney diseases at the primary health care level; a systematic review

**DOI:** 10.12861/jrip.2014.20

**Published:** 2014-07-01

**Authors:** Chaudhary Muhammad Junaid Nazar, Tiffany Billmeier Kindratt, Syed Muhammad Ahtizaz Ahmad, Manzoor Ahmed, John Anderson

**Affiliations:** ^1^Department of Medicine, University of Buckingham, Ealing Hospital, NHS Trust, London, UK; ^2^Department of Family and Community Medicine, University of Texas Southwestern Medical Center, Dallas, USA; ^3^Department of Community Medicine, Nawaz Sharif Medical College, Gujarat, Punjab, Pakistan; ^4^Department of Rural Health Development Program, Rural Area Development Council, Punjab, Pakistan; ^5^Division of Medical Education, Postgraduate Medicine Brighton & Sussex Medical School University of Brighton, Brighton, UK

**Keywords:** Chronic kidney disease, Renal transplantation, Dialysis, Established renal failure, Renal replacement therapy, Primary health care

## Abstract

**Background:** Chronic kidney disease (CKD), a major global public health problem, has been recognized as one of the eleven important causes of death. This review explores a wide range of barriers related to patients and health systems involved in controlling the prevalence of CKD at the primary health care level.

**Patients and Method:** Electronic databases including PubMed/Medline, Cumulative Index to Nursing and Allied Health (CINAHL), Entrez, British Medical Journal (BMJ), EBSCO host, Cochrane and Google scholar were searched for the data published from 2000 to 2010 using MeSH terms such as ‘chronic kidney diseases’, ‘renal transplantation’, ‘complications’, ‘health care services’, ‘acute renal failure’. After screening 587 abstracts, a total of 10 studies were selected for systematic review. Developed countries such as the United Kingdom, the USA and other European countries were reviewed in order to identify the barriers associated with CKD practice at the primary health care level. The reasons for the failure of services at the primary health care level were categorized. A pre-defined protocol was used for data extraction and content appraisal.

**Results:** At the primary health care level, the major barriers associated with CKD include the late referral of patients to nephrologists, old age, presence of several co-morbidities, lack of education and awareness among ethnic minorities, difficulty in communication between primary health care professionals, and the shortage of multi-disciplinary care team at dialysis centers. Additionally, factors such as drug-drug interaction during treatment, lack of anemia-management during dialysis, hypertension, and depression in CKD patients also act as important barriers in CKD care at the primary health care level.

**Conclusion:** The knowledge and awareness about CKD management is lacking. Therefore, educational intervention is essential for patients as well medical personnel. Also, a multidisciplinary care team is essential for the complex management of CKD due to associated co-morbidities.

**Implication for health policy/practice/research/medical education:**

Systematic review identified a wide range of barriers related to patients and health system involved in controlling the prevalence of CKD at the primary health care level. Lack of training and education of the primary health care is an important barrier for CKD and perhaps the most promising protective strategy for the government that can helps in early diagnosis of the kidney patients.

## Introduction

Chronic kidney disease (CKD is one of the leading causes of death and an emerging global public health problem. Multiple risk factors such as diabetes, hypertension, hyperlipidaemia and smoking play an important role in the progression of CKD (1). There are 15.4 million diagnosed CKD patients in the United Kingdom. In England, by the year 2025, nearly 42% rise in the population aged 65 or above, is expected to raise the population with at least one chronic condition by approximately 3 to 18 million (2). In the United States, 339 new cases of end-stage renal disease (ESRD) were reported per one million people in 2004. The total expenditure incurred on ESRD was 18.5 billion USD, out of which 16.3 billion dollars were spent exclusively on dialysis. For every patient, the cost of dialysis was reported to be USD 66,650 per year (3). An improved understanding of the implications of CKD will help in its prevention by supporting the behavioral and lifestyle changes. Therefore, CKD poses healthcare burden, not only in terms of finances but also in terms of health care personnel (2-4).

The use of renal replacement therapy either by dialysis or transplantation has become more common with the increased diagnosis of ESRD. Kidney transplantation promises an improved quality of life in comparison to dialysis and is thus a preferred treatment option (4). But the shortage of kidney transplant is well recognized globally. Treating established renal failure (ERF), a relatively rare condition, through dialysis or transplantation is an expensive option (1-5). In the United Kingdom, the number of patients undergoing renal replacement therapy (RRT) is increasing rapidly and is estimated to cost more than 2% of the total National Health Service (NHS) budget (2). Therefore, health education and utilization of primary health care services are essential preventive measures to modify a risk factor (1-4). The late referral of ESRD patients to a nephrologist leads to the poor prognosis and clinical outcomes, besides the high cost of treatment. The service needs to be more patient-oriented, giving people various choices for their disease management. Patients opting for self-care at home must be presented with various options, including the provision for hemodialysis at home, as recommended by the National Institute for Health and Clinical Excellence (2). Besides addressing the need for varied services in detecting CKD, more attention should be given to recognition and management of acute kidney injury at the primary health care level. Innovative changes should be made through monitoring of the services in order to ensure that resources are being used to the best effect and that patients get the best outcome.

This research is aimed at identifying and systematically reviewing evaluated studies on strategies for controlling the CKD prevalence. The issues addressed in this review include causes of increase in the prevalence of CKD every year, recognizing barriers for successful CKD care at the primary health care level, and to determine why governmental preventive strategies or policies are failing to control CKD, and finally to draw conclusions about the effectiveness of the interventions available to reduce the prevalence of CKD.

## Methodology for systematic review

### Criteria for inclusion and exclusion of research articles

The articles included in this study were original publications of randomized controlled trials, cohort or case-control studies, both qualitative and quantitative, where the primary aim of the study was to 1) either diagnose CKD or determine the risk factors of CKD or study the long-term effects of CKD; 2) evaluate governmental plans for renal services and strategies to reduce the CKD risk factors like hypertension, diabetes mellitus and their prevalence in society; 3) review primary health care services involving different interventions like education and training; and 4) assess studies published in English. Studies published from 2000-2010 were considered and the duplicate studies performed on the same set of patients were excluded. Studies from developing and under-developed countries were excluded due to the paucity of literature. The articles related to ESRD care were also excluded as our research was focused on the primary renal health care.

### Types of participants

The participants involved in studies were patients exhibiting stages 2-5 of the CKD and its associated co-morbidities such as cardiovascular diseases, hypertension and patients on renal replacement therapy. Also, renal health care personnel such as doctors, pharmacists, nurses, nutritionists and technical supporters were involved as they strongly influenced CKD treatment strategies. The CKD patients involved in the studies were considered regardless of their race or ethnicity.

### Types of intervention

We evaluated different types of intervention strategies to reduce the risk factors for the CKD prevalence at the primary health care level. These included better education and training of nurses for fluid management, vascular access and nutrition in dialysis patients; multi-disciplinary care conferences for doctors; blind audit of staff performance; an early referral of patients to nephrologists; and early detection of CKD through improved diagnostic services. Other effective interventions could be procedural, such as implementation of polices in a facility; attitudinal, such as staff morale; personal, such as staff communication; structural, such as the layout of facilities; and belief-oriented, such as belief in the role of the facilities-based health maintenance. All these services must be included at the primary health care level, for better outcomes in CKD patients.

### Search methods for identification of studies

**Figure 1:**
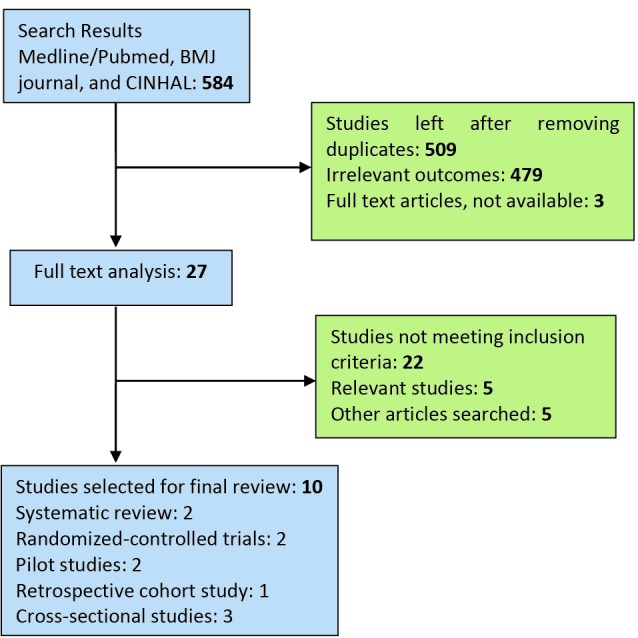
Flow chart showing the search, the major reasons for the exclusion of studies and the final number of articles included in the study.

Electronic databases such as Medline/PubMed, BMJ journals, CINAHL and Cochrane Collaboration were searched extensively using MeSH terms such as ‘chronic kidney diseases’, ‘renal transplantation’, ‘complications’, ‘health care services’, ‘acute renal failure’, along with the other terms such as prognosis, mortality, outcomes and diagnosis. Data from the Department of Health (DOH) and the National Health Services (NHS), especially different governmental policies regarding the services for kidney patients, were accessed in collaboration with the library staff at the University of Bedfordshire. Overall, 10 studies involving 13 trials were selected for this review. On searching Medline and CINAHL, 584citations were obtained, out of which only 509 were left after adjusting for duplicates. Of these, 479 studies did not meet the exclusion criteria and were excluded. Three additional studies were discarded as their full texts were unavailable or they could not be reasonably translated into English. The full texts of the remaining 27 citations were carefully examined, out of which only five studies meeting the inclusion criteria were included in the systematic review. Additional five studies meeting the inclusion criteria were identified by checking the references cited in these papers. No unpublished relevant studies were included in the review (Figure 1).

### Assessment of the selected articles

AMSTAR(A MeaSurement Tool to Assess systematic Reviews), was used to assess the methodological quality of the articles selected for this study. All ten articles selected for the final review were found to comply with all the eleven criteria mentioned in the AMSTAR checklist (Table 1).

**Table 1:** An AMSTAR-based quality assessment of selected articles

**S. No.**	**AMSTAR Checklist**	**The articles selected for the systematic review**									
		**1**	**2**	**3**	**4**	**5**	**6**	**7**	**8**	**9**	**10**
1	Was an 'a priori' design provided?	Yes	Yes	Yes	Yes	Yes	Yes	Yes	Yes	Yes	Yes
2	Was there duplicate study selection and data extraction?	Yes	Yes	Yes	Yes	Yes	Yes	Yes	Yes	Yes	Yes
3	Was a comprehensive literature search performed?	Yes	Yes	Yes	Yes	Yes	Yes	Yes	Yes	Yes	Yes
4	Was the status of publication (i.e. grey literature) used as an inclusion criterion?	Yes	Yes	Yes	Yes	Yes	Yes	Yes	Yes	Yes	Yes
5	Was a list of studies (included and excluded) provided?	Yes	Yes	Yes	Yes	Yes	Yes	Yes	Yes	Yes	Yes
6	Were the characteristics of the included studies provided?	Yes	Yes	Yes	Yes	Yes	Yes	Yes	Yes	Yes	Yes
7	Was the scientific quality of the included studies assessed and documented?	Yes	Yes	Yes	Yes	Yes	Yes	Yes	Yes	Yes	Yes
8	Was the scientific quality of the included studies used appropriately in formulating conclusions?	Yes	Yes	Yes	Yes	Yes	Yes	Yes	Yes	Yes	Yes
9	Were the methods used to combine the findings of studies appropriate?	Yes	Yes	Yes	Yes	Yes	Yes	Yes	Yes	Yes	Yes
10	Was the likelihood of publication bias assessed?	Yes	Yes	Yes	Yes	Yes	Yes	Yes	Yes	Yes	Yes
11	Was the conflict of interest included?	Yes	Yes	Yes	Yes	Yes	Yes	Yes	Yes	Yes	Yes

### Data extraction

The data extraction sheet (Appendix 1), study design, methodology, findings, discussion and the conclusions were refined accordingly. First, an author extracted the data from included studies, and then the other author evaluated it thoroughly. All discrepancies were resolved through consensus.

### Data generation

This systematic review involves studies with various study designs, methodologies and findings. Each research work was thoroughly reviewed and a summary was prepared. The full-text article of each potentially relevant citation was retrieved and independently evaluated. In duplicate studies involving the same set of patients, the study with the larger set of patients was included. Any discrepancies were sorted out through consensus (Table 2).

**Table 2:** he selected articles are mentioned in the table with the author’s name, the year of publication, study design, outcome measures and sample size.

**S. No.**	**Author & Year**	**Title**	**Type of the research**	**Study design**	**Sample population**	**Result**
1	Desai *et al.* (5)	Identifying best practices in dialysis care: Results of cognitive interviews and a national survey of dialysis providers	Systematic review and cognitive interview involving focus group	Best practices in dialysis were discovered through a staged process which included methodical review, perceptive interviews, and a national “virtual focus group” of dialysis providers.	Phase 1: systematic review and cognitive interview with the focus group (nurses, doctors and medical staff) Phase 2: areas of agreement and disagreement on the issue Phase 3: validation of the best practices identified.	Significantly higher mortality and increased early hospitalization of CKD subjects who were referred late to nephrologists as compared with earlier-referred subjects.
2	Navaneethan *et al.* (6)	A systematic review of patient and health system characteristics associated with the late referral in chronic kidney disease	A systematic review	Abstracts of 256 articles and 18 observational studies were selected		Primary-care physicians and nephrologists should engage in multi-spectral, collaborative efforts for patient education and enhanced physician awareness for improved CKD patient care.
3	Odden *et al.* (7)	Depression, stress, and quality of life in person with CKD: The heart and soul study	A Cross-setional survey	Cross-sectional study		The quality of life is impaired in the subjects with moderate CKD.
4	Stemer *et al. *(16)	Evaluation of risk factors management of patients treated on an internal nephrology ward	A pilot study	Medical charts of patients treated on a single internal nephrology ward were retrospectively assessed using pre-defined data collection form.	102 randomly selected medical histories were used to carry out pilot study.	Attention should be paid to the risk factors associated with the management of drug-drug interaction and screening procedures used for CKD diagnosis.
5	Guessous *et al. *(13)	Low documentation of chronic kidney disease among high-risk patients in a managed care population: A retrospective cohort study	A retrospective cohort study	Participants having GFRs 60- 365 days were <90 ml/min during 1999-2006. The analysis included participants with eGFR 10-59 ml/min/1.73 m^2^.	50,438 CKD patients were selected within the overall KPG CKD group. 20% (10,266) were eligible for the current study.	The frequency of CKD documentation increased with the presence of hypertension and/or type-2 diabetes.
6	Leehey *et al.* (8)	Aerobic exercise in obese diabetic patients with chronic kidney disease	Arandomized and controlled pilot study	After physical examination and ECG, the participants took a symptom-limited treadmill exercise stress test. Negative subjects were given questionnaires, nutritional assessment and laboratory tests. The eligible patients were randomized to the exercise or control group. This test was repeated after 6 weeks.	The study involved 20 subjects from the renal outpatient clinic with type-2 diabetes, obesity (BMI >30 Kg/m^2^), and stage 2-4 CKD (eGFR15-90 ml/min/1.73 m^2^) with persistent protein urea, i.e. urine protein/creatinine >200 mg/g for ≥3 months).	The obese diabetic patients with CKD may benefit from exercise training.
7	Manns *et al. *(10)	The impact of education on CKD patients’ plans to initiate dialysis with self-care dialysis: A randomized trial	A randomized trial		70 patients receiving care in the renal unit were randomized.	An educational intervention can increase the number of patients opting for self-care dialysis.
8	Hallan *et al. *(14)	Screening strategies for chronic kidney disease in general population	Cross-sectional survey	Eight year follow-up of a cross-sectional health survey	65,604 people (70.6 %) of all adults aged ≥20 in the country.	Screening people for hypertension, diabetes mellitus, or age >55 was the most effective in detecting CKD.
9	Ahmed (15)	Current chronic kidney disease practice patterns in the UK	A pilot study	A pre-piloted questionnaire was sent to all 72 renal units in the UK.		Current services need to be re-designed to deal with the expected rise in the referral of CKD patients in UK.
10	Lenzo *et al.* (9)	Barriers to successful care for chronic kidney disease	Cross-sectional survey	Laboratory parameters for serum calcium, phosphate,intact PTH, albumin, bicarbonate, and hemoglobin were obtained from chart review.	268 patients with clinical appointment were selected.	Raising awareness of CKD and K/DOQI goals among primary care providers, early referral to a nephrologist, studying socio-economic and cultural barriers, and both patient and physician education are critical to improve CKD care.

## Results

The results of the review have been grouped into various categories (Table 3). Seven studies categorized as personal involved lack of education, awareness and exercise, and old age, which were considered as the most common barriers (5-10). This is also consistent with other findings which revealed that CKD is more prevalent in males and ethnic minorities (11,12).

**Table 3:** The results of the review.

**Barriers in CKD care at the primary health care level**	**References**
**Personal** • Education • Awareness • Old age • Lack of exercise	Desai *et al*. (5); Navaneethan *et al*. (6); Odden *et al*. (7); Leehey *et al*. (8); Lenzo *et al*. (9); Manns *et al*. (10)
**Procedural** • Lack of Training • Proper diagnostic tools • Late referral	Guessous *et al*. (13); Hallan *et al*. (14); Lenzo *et al*. (9); Desai *et al*. (5)
**Attitudinal** • Lack of communication • Belonging to ethnic minorities • Staff morale	Navaneethan *et al*. (6)
**Biological** • Pharmacological dependency	Stemer *et al*. (16)
**Structural** • Lack of multidisciplinary team	Stemer *et al*. (16), Desai *et al*. (5)
**Environmental** • Work load on nephrologists • Quality of life	Ahmed (15), Odden *et al*. (7)

The second most popular category was procedural, which included five studies showing lack of training of medical professionals such as doctors, nurses, primary health care physicians and highlights the need for re-designing of better diagnostic tools for early detection of CKD (5,9,13,14). All these factors lead to the late detection or the late referral of CKD patients to the nephrologists, ensuing severe consequences (6,11).

Environmental barriers included heavy workload on health care professionals who lack time to counsel patients on multiple aspects of CKD and associated risk factors (15). Odden *et al.* suggested that CKD patients with coronary artery disease have a poor quality of life due to constant depression and stress (7). Attitudinal barriers include lack of communication between the primary health care physicians and patients belonging to ethnic minorities. Such patients are less educated and unaware about the treatment strategies, causing the poor prognosis of CKD (6). The lack of staff morale and communication among the dialysis team members also acts as an important attitudinal barrier (5). Pharmacological dependency acts as a biological barrier. Clinical pharmacy services can help in risk factor management and systematic medication reviews of potential drug-drug interactions (16). Lack of documentation by primary and subspecialty care providers results in the poor diagnosis of patients (13). Structural barriers include improper layout of facilities in the dialysis centers and internal nephrology wards with a wide variation in services. Owing to its associated co-morbidities, the CKD management is quite complex. Therefore, a multi-disciplinary team involving nutritionists, pharmacists, technicians, primary health care physicians, and vascular access surgeons must be available in all wards (5,16).

## Discussion

### Findings

This systematic review consolidates findings from 10 different research articles, involving diverse methodologies selected from the USA, Canada, UK, and other European countries. Evidently, the barriers at the primary health care level include lack of communication between the physicians and patients; lack of awareness, education and training of nurses; and the heavy workload on nephrologists. Other barriers include late referrals to the nephrologists by general physicians; the late diagnosis of CKD; need for re-designing routine screening strategies; old age and the poor quality of life in patients belonging to ethnic minorities; and associated multiple co-morbidities. The government has initiated various programs, such as ABLE (A Better Life through Education and Empowerment), to raise the awareness of CKD. Chronic renal failure (CRF), an irreversible condition, when becomes established leads to an end-stage renal disease (ESRD), for which the ultimate treatment is dialysis or transplantation of a kidney (2).

## Limitations of the Study

This systematic review had certain limitations. Firstly, eligibility was limited to English language articles, articles published after 2000, and to studies conducted only in the developed countries. Secondly, the majority of the studies identified barriers associated with the primary health care level, preventing us to determine the factors associated with health hazards such as organ transplantation, delayed grafting, issues related with ESRD patients. Therefore, more research is needed to address these issues in future. Also, the intervention of secondary or tertiary health care levels was not considered for the control of CKD.

## Conclusion

This systematic review identified a wide range of barriers related to patients and health system involved in controlling the prevalence of CKD at the primary health care level. Lack of training and education of the primary health care is an important barrier for CKD care. Doctors must be educated about how to diagnose the CKD and how to write the prescription properly so that they are able to refer patients early to nephrologists for better treatment and prognosis. The standards or layout of the facilities in all the nephrology wards must be same. A multidisciplinary care team including doctors, surgeons, nurses, nutritionists and technicians is essential for the complex management of CKD due to associated co-morbidities such as diabetes, cardiovascular diseases, hypertension, etc. The knowledge and awareness about CKD management is lacking. Therefore, educational intervention for the patients is essential. Also, the complexity of pharmaceutical management of CKD necessitates proper medication of the patients, especially those undergoing dialysis. The CKD patients who are old and belong to ethnic minorities are less educated and, therefore, unable to communicate with primary health care physicians, which in turn leads to their late referral to nephrologists. Further research is needed in exploring several strategies for preventing the risk factors associated with CKD care at the primary health care level.

## Recommendations

The combinations of strategies for controlling CKD at the primary health care level among diabetic and ESRD patients need to be monitored continuously and redesigned for better results. Associated co-morbidities and major risk factors make the medical care of CKD-patients quite complex (3,6). The main consequence of CKD is not only the progression to dialysis or ESRD but also an increased risk of heart diseases, particularly in cases of diabetes and hyperlipidaemia (7). Thus, the evaluation of all these risk factors is essential for successful CKD management.

Moreover, the prevalence of CKD in the ethnic minorities necessitates their education using strategic means, such as during an election campaign when new policies are implemented (5). The patients with stage-3 CKD have significant renal impairment and poorly recognized renal failure, while the patients with stage-4 and stage-5 CKD exhibit renal anemia and osteodystrophy. At these stages, educating patients definitely plays an important role in their preparedness for the renal replacement therapy (7). A wide variation in the prevalence of the treated ESRD in the developed countries has led to a decrease in patients on renal replacement therapy and it is assumed that some patients are never identified or referred to nephrologists (13). Therefore, reporting or documentation of such patients should be introduced as soon as possible.

Furthermore, the focus of the primary care targets should not be limited just to the recognition and treatment of CKD but also to monitoring of the renal functions such as relative effects of delayed dialysis. The steps must be taken to identify patients who need early dialysis to prevent complications of CKD and cardiovascular deaths. Besides the relaxation of selection criteria for dialysis, the rising prevalence of diabetes and changing population also influence the true incidence of CKD. Despite of the fact, the evidence-based referral and management guidelines have been produced by the Renal Association and the Royal College of Physicians of London (2), which if followed properly, will help reduce the incidence of preventable mortalities from CKD.

Overall, the review proposes that different types of interventions can be applied as strategies for controlling CKD prevalence at the primary health care level. To reduce the barriers and increase the efficacy of the services at a facility, highly trained staff and frequency of dialysis physician visits must be increased. Conferences must be held regularly to educate the nurses and doctors. Highly skilled and experienced staff must be arranged to deal with the patients suffering from the established renal failure and patients must have their regular check-up and follow-ups.

## Authors’ contributions

CMJN completed the whole article. AML and SMAA played an important part in formatting. JA AND KTB review and revise the whole.

## Conflict of interests

The authors declared no competing interests.

## Ethical considerations

Ethical issues (including plagiarism, data fabrication, double publication) have been completely observed by the author.

## Funding/Support

It is self-financed.
